# A New Deep Learning Model for Fault Diagnosis with Good Anti-Noise and Domain Adaptation Ability on Raw Vibration Signals

**DOI:** 10.3390/s17020425

**Published:** 2017-02-22

**Authors:** Wei Zhang, Gaoliang Peng, Chuanhao Li, Yuanhang Chen, Zhujun Zhang

**Affiliations:** State Key Laboratory of Robotics and System, Harbin Institute of Technology, No. 92 Xidazhi Street, Harbin 150001, China; zw1993@hit.edu.cn (W.Z.); li_chuanhao@126.com (C.L.); cyh.wne@gmail.com (Y.C); zhangzhujun36@126.com (Z.Z.)

**Keywords:** intelligent fault diagnosis, convolutional neural networks, domain adaptation, anti-noise

## Abstract

Intelligent fault diagnosis techniques have replaced time-consuming and unreliable human analysis, increasing the efficiency of fault diagnosis. Deep learning models can improve the accuracy of intelligent fault diagnosis with the help of their multilayer nonlinear mapping ability. This paper proposes a novel method named Deep Convolutional Neural Networks with Wide First-layer Kernels (WDCNN). The proposed method uses raw vibration signals as input (data augmentation is used to generate more inputs), and uses the wide kernels in the first convolutional layer for extracting features and suppressing high frequency noise. Small convolutional kernels in the preceding layers are used for multilayer nonlinear mapping. AdaBN is implemented to improve the domain adaptation ability of the model. The proposed model addresses the problem that currently, the accuracy of CNN applied to fault diagnosis is not very high. WDCNN can not only achieve 100% classification accuracy on normal signals, but also outperform the state-of-the-art DNN model which is based on frequency features under different working load and noisy environment conditions.

## 1. Introduction

Rolling element bearings are the core components in rotating mechanisms, whose health conditions, for example, the fault diameters in different places under different loads, could have enormous impact on the performance, stability and life span of the mechanism. The most common way to prevent possible damage is to implement a real-time monitoring of vibration when the rotating mechanism is in operation. With the vibration signals under different conditions collected by the sensors, intelligent fault diagnosis methods are applied to recognize the fault types [1,2,3]. Common intelligent fault diagnosis methods can be divided into two steps, namely, feature extraction and classification [4,5]. The vibration signals collected from machines are raw temporal signals which contain the useful information of the machine, as well as useless noise. Therefore, it’s necessary to find a way to extract useful features that represent the intrinsic information of the machine. Common signal processing techniques used to extract the representative features from the raw signal include time-domain statistical analysis [6], wavelet transformation [7], and Fourier spectral analysis [8]. Usually after feature extraction, a feature selection step will be implemented to get rid of useless and insensitive features, and reduce the dimensions for the sake of computational efficiency. Common dimension reduction methods include principal component analysis (PCA) [9], independent component analysis (ICA) [10], and feature discriminant analysis. With the useful features extracted and selected from raw signals, the last step is to train classifiers like k-nearest neighbor (KNN) [11], artificial neural networks(ANN), also known as Multi-layer Perceptron (MLP) [12,13], or support vector machine (SVM) [14] with these features. After training, the classifiers should be tested on test samples to see if they can generalize well unseen signal samples.

In recent years, Huang et al. proposed a genetic algorithm-based SVM (GA-SVM) model that can determine the optimal parameters of SVM with high accuracy and generalization ability [15]. In [16], continuous wavelet transform was used to overcome the shortcomings of the traditionally used Fourier transform, like not being able to tell when a particular event took place, and then SVM is used as the classifier to analyze frame vibrations. MLP, known for its capability to learn features with complex and nonlinear patterns, has also been a very common classifier used in fault diagnosis. Amar proposed a fault diagnosis method which uses a preprocessed FFT spectrum image as input of ANN. The FFT spectrum image generated from raw vibration signal is first averaged using a 2D averaging filter and then converted to binary image with appropriate threshold selection [17]. In [18], the discrete wavelet transform is used for feature extraction and an artificial neural network is used for classification.

In recent years, with the surging popularity of deep learning as a computational framework in various research fields, some papers have tried to use convolutional neural networks [19] to diagnose the fault of mechanical parts. CNNs have two main features: weights sharing and spatial pooling, which makes it very suitable for computer vision applications whose inputs are usually 2D data, but it has also been used to address natural language processing and speech recognition tasks whose inputs are 1D data [20,21]. Therefore, in fault diagnosis problem, the inputs of CNNs can be either 2D, e.g., frequency spectrum image, or 1D, e.g., time-series signal or spectral coefficients. Janssens et al. proposed a CNN model for rotating machinery conditions recognition whose input is DFT of two lines of signals collected from two sensors placed perpendicular to each other [22]. The model has one convolutional layer and one fully connected layer, and on top of the network is a softmax layer for classification into four categories. In [23], a hierarchical adaptive deep convolutional neural network. The model has two hierarchically arranged components: a fault determination layer and a fault size evaluation layer. In [24], the inputs of the CNN model for motor fault detection is 1D raw time series data, which successfully avoids the time-consuming feature extraction process. In [25], the proposed model also uses 1D vibration signal as input. It can perform real-time damage detection and localization. With raw vibration signals fed directly into the network, the optimal damage-sensitive features are learned automatically. In [26], we proposed a CNN model with two convolutional layers to diagnose the faults of bearings with a huge number of training data.

Though many of the works mentioned above have achieved pretty good results, there is still plenty of room for improvement. For example, in many studies, the classifier was trained with a very specific type of data, which means it may achieve high accuracy on similar data while performing poorly with another type. This may be caused by the wrong presentative features extracted from the raw signals. Besides, when analyzing a highly complex system, the choice of suitable feature functions requires considerable machinery expertise and abundant mathematical knowledge. In other hands, because of the manual feature extraction and selection, the accuracy of the diagnostic result would not be stable when dealing with different data. Therefore, some studies proposed that the classifier should have the ability to classify the data from the raw signal directly, without feature extraction or manual selection [24,27]. In other words, the classifier should have the ability to process the raw signal automatically and adaptively and extract the presentative features more precisely. To sum up, there are three main problems existing in intelligent fault diagnosis.

First, although many methods can achieve good results in fault diagnosis, few of them work directly on raw temporal signals. Most of the algorithms have the same classifiers, such as SVM and MLP, etc. These paper mainly focuses on improving feature representation and extraction.

Second, many diagnosis methods have poor domain adaptation ability. It is not uncommon to find that a classifier trained with data from one working load fails to classify samples obtained from another working load properly.

Third, few algorithms perform well under noisy environment conditions. Various pre-processing methods are used to remove noise and to improve the classification accuracy, but few methods can classify the signals directly on raw noisy signals with high accuracy.

**Figure 1:**
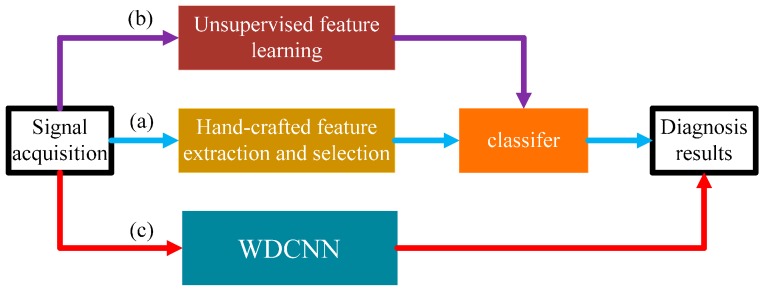
Three intelligent fault diagnosis frameworks: (**a**) traditional method; (**b**) features extracted by unsupervised learning [27]; (**c**) the proposed method.

In order to address the problems above, in this paper, we proposed a method named Deep Convolution Neural Networks with Wide first-layer kernels (WDCNN). The contributions of this paper are summarized below:

(1) We propose a novel and simple learning framework, which works directly on raw temporal signals. A comparison with traditional methods that require extra feature extraction is shown in Figure 1.
(2) This algorithm itself has strong domain adaptation capacity, and the performance can be easily improved by a simple domain adaptation method named AdaBN.
(3) This algorithm performs well under noisy environment conditions, when working directly on raw noisy signals with no pre-denoising methods.
(4) We try to explore the inner mechanism of WDCNN model in mechanical feature learning and classification by visualizing the feature maps learned by WDCNN.

The remainder of this paper is organized as follows: a brief introduction of CNN is provided in Section 2. The intelligent diagnosis method based on WDCNN is introduced in Section 3. Some experiments are conducted to evaluate our method against some other common methods. After this, discussion about the results of the experiments is presented in Section 4. We draw the conclusions and present the future work in Section 5.

## 2. A Brief Introduction to CNN

The architecture of CNN is briefly introduced in this section, and more details on CNN can be found in [19]. The convolutional neural network is a multi-stage neural network which is composed of some filter stages and one classification stage. The filter stage is designed to extract features from the inputs, which contains two kinds of layers, the convolutional layer and the pooling layer. The classification stage is a multi-layer perceptron, which is composed of several fully-connected layers. The function of each type of layer will be described below.

### 2.1. Convolutional Layer

The convolutional layer convolves the input local regions with filter kernels and then followed by the activation unit to generate the output features. Each filter uses the same kernel to extract the local features of the input local region, which is usually referred to as weight-sharing in the literature. One filter corresponds to one frame in the next layer, and the number of frames is called the depth of this layer. We use $K_{i}^{l}$ and $b_{i}^{l}$ to denote the weights and bias of the *i*-th filter kernel in layer *l*, respectively, and use $x^{l}\left(j\right)$ to denote the *j*-th local region in layer *l*. Therefore, the convolution process is described as follows:

(1) $$y_{i}^{l+1}\left(j\right)=K_{i}^{l}*x^{l}\left(j\right)+b_{i}^{l}$$

where the notation * computes the dot product of the kernel and the local regions, and $y_{i}^{l+1}\left(j\right)$ denotes the input of the *j*-th neuron in frame *i* of layer *l* + 1.

### 2.2. Activation Layer

After the convolution operation, activation function is essential. It enables the network to acquire a nonlinear expression of the input signal to enhance the representation ability and make the learned features more dividable. In recent years, Rectified Linear Unit (ReLU) was widely used as activation unit to accelerate the convergence of the CNNs. ReLU makes the weights in the shallow layer more trainable when using back-propagation learning method to adjust the parameters. The formula of ReLU is described as follows:

(2) $$a_{i}^{l+1}\left(j\right)=\;f\left(y_{i}^{l+1}\left(j\right)\right)=\max\left\{0,y_{i}^{l+1}\left(j\right)\right\}$$

where $y_{i}^{l+1}\left(j\right)$ is the output value of convolution operation and $a_{i}^{l+1}\left(j\right)$ is the activation of $y_{i}^{l+1}\left(j\right).$

### 2.3. Pooling Layer

It is common to add a pooling layer after a convolutional layer in the CNN architecture. It functions as a down-sampling operation which reduces the spatial size of the features and the parameters of the network. The most commonly used pooling layer is max-pooling layer, which performs the local max operation over the input features, to reduce the parameters and obtain location-invariant features. The max-pooling transformation is described as follows:

(3) $$P_{i}^{l+1}\left(j\right)=\underset{\left(j-1\right)W+1\leq t\leq jW}{\max}\left\{q_{i}^{l}\left(t\right)\right\}$$

where $q_{i}^{l}\left(t\right)$ denotes the value of *t*-th neuron in the *i*-th frame of layer *l*, t∈[(j−1)W+1,jW], *W* is the width of the pooling region, and $P_{i}^{l+1}\left(j\right)$ denotes the corresponding value of the neuron in layer *l* + 1 of the pooling operation.

### 2.4. Batch Normalization

The batch normalization [28] layer is designed to reduce the shift of internal covariance and accelerate the training process of the deep neural network. The BN layer is usually added right after the convolutional layer or fully-connected layer and before the activation unit. Given the *p*-dimension input to a BN layer $x=\left(x^{\left(1\right)},\ldots ,x^{\left(p\right)}\right)$, the transformation of the BN layer is described as follows:

(4) $$\begin{array}{c} {\hat{x}}^{\left(i\right)}=\frac{x^{\left(i\right)}-E\left[x^{\left(i\right)}\right]}{\sqrt{Var\left[x^{\left(i\right)}\right]}}\\ y^{\left(i\right)}=\gamma^{\left(i\right)}{\hat{x}}^{\left(i\right)}+\beta^{\left(i\right)} \end{array}$$

where $y^{\left(i\right)}$ is the output of one neuron response, $\gamma^{\left(i\right)}$ and $\beta^{\left(i\right)}$ are the scale and shift parameters to be learned, respectively.

The first step is to standardize feature in each dimension independently, which helps to accelerate convergence. Then $\gamma^{\left(i\right)}$ and $\beta^{\left(i\right)}$ are used to scale and shift each normalized feature, to ensure the transformation inserted in the network can represent the identity transform. In other words, $\gamma^{\left(i\right)}$ and $\beta^{\left(i\right)}$ are used to restore the representation power of the network.

## 3. Proposed WDCNN Intelligent Diagnosis Method

As mentioned in Section 1, CNN has already been applied to fault diagnosis. However, these models fail to achieve a higher performance than traditional methods. Most of the models are not deep enough, for example, the model used in [24] only has three convolutional layers, which makes it hard to obtain the high nonlinear expression of the input signal. Therefore, in order to give the kernels in the third layer a large enough receptive field to capture low frequency features, e.g., periodical changes in the signal, the size of the convolutional kernels cannot be too small. On the other hand, in order to preserve local features, the convolutional kernels cannot be too large. As a compromise, these models use middle size kernels.

**Figure 2:**
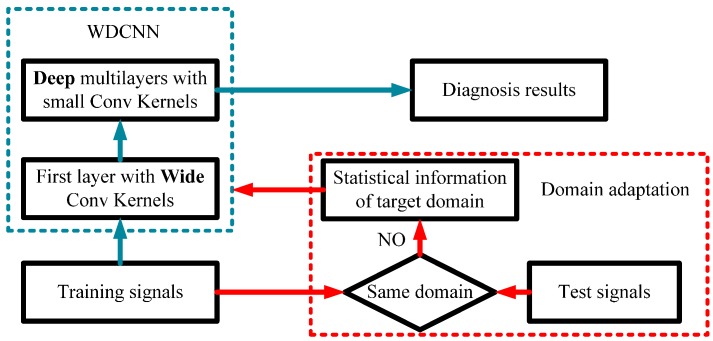
The overall framework of proposed WDCNN with AdaBN domain adaptation.

Besides, 1-D vibration signals are different from 2D images. For a 224 × 224 image in Imagenet, the VGGnet [29] performs well with all small 3 × 3 convolutional kernels. However, for a 2048 × 1 vibration signals, designing a model with all small 3 × 1 kernels is unrealistic. This will result in a very deep network, making it very hard to train. In addition, small kernels at the first layer are easily disturbed by high frequency noise common in industrial environments. Therefore, to capture the useful information of vibration signals in the intermediate and low frequency bands, we first used wide kernels to extract features, and then use successive small 3 × 1 kernels to acquire better feature representation, hence the model is deeper than the former CNN method. That’s why we name our model WDCNN, with W denoting wide kernels in the first layer and D denoting the deep structure. The overall framework of proposed WDCNN with AdaBN domain adaptation is shown in Figure 2. Details of each parts are elaborated in the following subsections.

### 3.1. Architecture of the Proposed WDCNN Model

**Figure 3:**
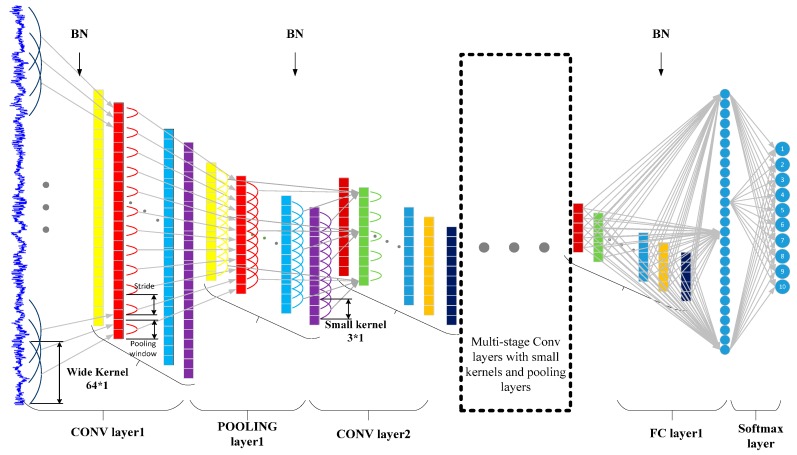
Architecture of the proposed WDCNN model.

As shown in Figure 3, the input of the CNN is a segment of normalized bearing fault vibration temporal signals. The first convolutional layer extracts features from the input raw signal without any other transformation. The overall architecture of proposed WDCNN model is the same as that of normal CNN models. It is composed of some filter stages and one classification stage. The major difference is that, in the filter stages, the first convolutional kernels are wide, and the following convolutional kernels are small (specifically, 3 × 1). The wide kernels in the first convolutional layer can better suppress high frequency noise compared with small kernels. Multilayer small convolutional kernels make the networks deeper, which helps to acquire good representations of the input signals and improve the performance of the network. Batch normalization is implemented right after the convolutional layers and the fully-connected layer to accelerate the training process.

The classification stage is composed of two fully-connected layers for classification. In the output layer, the softmax function is used to transform the logits of the ten neurons to conform the form of probability distribution for the ten different bearing health conditions. The softmax function is described as:

(5) $$q\left(z_{j}\right)=\frac{e^{z_{j}}}{\sum_{k}^{10}e^{z_{k}}}$$

where **z***_j_* denotes the logits of the *j*-th output neuron.

### 3.2. Training of the WDCNN

The architecture of WDCNN is designed to take advantage of the 1D structure of the input signals. Details about the architecture of WDCNN can be found in Section 4.2. Major structural differences between traditional 2D CNN and 1-D CNN like the proposed WDCNN are the use of 1-D kernels and 1D feature maps. Therefore, different from 2D convolution (conv2D) and lateral rotation (rot180) during backpropagation, here we have 1D convolution (conv1D) and reverse. In this part, we will elaborate the training process of WDCNN using back propagation algorithm.

The loss function of our CNN model is the cross-entropy between the estimated softmax output probability distribution and the target class probability distribution. No regularization term is added to the loss function, considering Batch Normalization already has a similar effect as regularization. Let *p*(*x*) denote the target distribution and *q*(*x*) denote the estimated distribution, so the cross-entropy between *p*(*x*) and *q*(*x*) is:

(6) $$Loss=H\left(p,q\right)=-\underset{x}{\sum }p\left(x\right)\log q\left(x\right)$$

The fully connected layers are identical to the layers in a standard multilayer ANN. Specifically, let $\delta^{l+1}$ be the error for the *l* + 1 layer in the fully connected network with a cost function **H**, where (**W,b**) are the parameters. Then the error for the *l* layer is computed as:

(7) $$\delta^{l}=({\left(W^{l}\right)}^{T}\delta^{l+1})•f^{\prime }(z^{l})$$

where “●” denotes the element-wise product operator.

The iteration of gradient descent updates the parameters as follows:

(8) $$\begin{array}{c} W_{ij}^{l}=W_{ij}^{l}-\alpha \frac{\partial H}{\partial W_{ij}^{l}}\\ b_{i}^{l}=b_{i}^{l}-\alpha \frac{\partial H}{\partial b_{i}^{l}} \end{array}$$

where *α* is the learning rate.

Pooling layer down-samples statistics to obtain summary statistics from the training set. Down-sampling is an operation like convolution, however *g* is applied to non-overlapping regions.

Let *m* be the size of pooling region, *x* be the input, and *y* be the output of the pooling layer. The term downsample(*f,g*)[*n*] denotes the *n*-th element of downsample(*f,g*):

(9) $$y_{n}=\mathrm{downsample}(x,g)[n]=g(x_{(n-1)m+1:nm})$$

Here we use Max Pooling, so *g*(*x*) = max(*x*).

Backpropagation in the pooling layer reverses the above equation, which means error signals for each example are computed by up-sampling. In max pooling, the unit which was the max at forward feed receives all the error at backward propagation:

(10) $$\begin{array}{c} \frac{\partial g}{\partial x_{i}}=\{\begin{array}{cc} 1 & if\;x_{i}=\max(x)\\ 0 & \mathrm{otherwise} \end{array}\\ {g^{\prime }}_{n}=\frac{\partial g}{\partial x_{(n-1)m+1:nm}} \end{array}$$

where $g_{n}^{\prime }$ is changeable depending on pooling region *n*:

(11) $$\delta_{(n-1)m+1:nm}^{\left(x\right)}=\delta_{n}^{\left(y\right)}{g^{\prime }}_{n}=\frac{\partial H}{\partial y_{n}}\frac{\partial y_{n}}{\partial x_{(n-1)m+1:nm}}=\frac{\partial H}{\partial x_{(n-1)m+1:nm}}$$

Finally, to calculate the gradient of the filter maps, we rely on the convolution operation again and flip the error matrix $\delta_{k}^{l}$ in the same way as we flip the filters in the convolutional layer:

(12) $$\begin{array}{c} \nabla_{W_{k}^{l}}H=\sum_{i=1}\left(a_{i}^{l})*\mathrm{flip}({\delta_{k}}^{l+1}\right)\\ \nabla_{b_{k}^{l}}H=\sum_{a,b}{\left({\delta_{k}}^{l+1}\right)}_{a,b} \end{array}$$

where *a^l^* is the input to the *l*-th layer. The operation “*” computes the valid convolution between *i*-th input in the *l*-th layer and the error of the *k*-th kernel. The flip results from derivation of delta error in Convolution Neural Network.

### 3.3. Domain Adaptation Framework for WDCNN

Domain adaptation is a realistic and challenging problem in fault diagnosis. It is hard to classify a sample from a working environment while the classifier is trained by the samples collected in another working environment. The working environment can be considered as a domain, so the domain in which we acquire labeled data and train our model is called source domain, and the domain in which we only obtain unlabeled data and test our model is named target domain. Then the problem above can be regarded as a domain adaptation problem.

**Figure 4:**
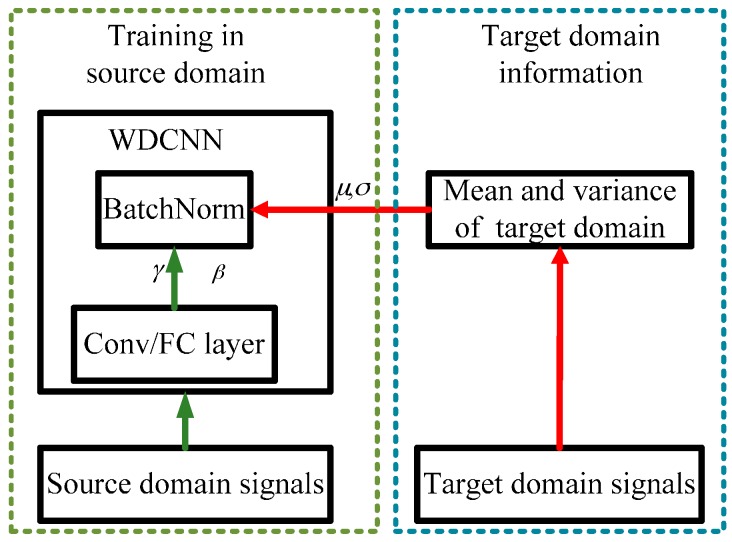
Domain adaptation framework for WDCNN.

In 2016, Li et al. proposed a simple method named Adaptive Batch Normalization (AdaBN) [30] to utilize BN to endow neural networks with good domain adaptation capacity. This algorithm can be easily combined with our WDCNN model because of the heavy use of BN in our model. The main problem existing in domain adaptation is the divergence of distribution between the target domain and source domain. AdaBN standardizes each sample by the statistics in the domain it belongs to instead of using the statistics of the source domain all the time. Its purpose is to ensure that each layer receives data complying with a similar distribution, regardless of the source domain or target domain. The framework of AdaBN is shown in Figure 4, and details of AdaBN for WDCNN is described in Algorithm 1.

**Algorithm 1** AdaBN for WDCNN  **Input:**Input of neuron *i* in BN layers of WDCNN for unlabeled target signal p, $x_{t}^{\left(i\right)}\left(p\right)\in x_{t}^{\left(i\right)}$,where $x_{t}^{\left(i\right)}=\{x_{t}^{\left(i\right)}\left(1\right),\ldots ,x_{t}^{\left(i\right)}\left(n\right)\}$ The trained scale and shift parameters $\gamma_{s}^{\left(i\right)}$ and $\beta_{s}^{\left(i\right)}$ for neuron *i* using the labeled source signals.  **output:**Adjusted structure of WDCNN  **For**Each neuron *i* and each signal *p* in target domain Calculate the mean and variance of all the samples in target domain: $\mu_{t}^{\left(i\right)}\leftarrow E[x_{t}^{\left(i\right)}]$ $\sigma_{t}^{\left(i\right)}\leftarrow Var[x_{t}^{\left(i\right)}]$ Calculate the BN output by: ${\hat{x}}_{t}^{\left(i\right)}\left(p\right)=\frac{x_{t}^{\left(i\right)}(p)-\mu_{t}^{\left(i\right)}}{\sigma_{t}^{\left(i\right)}}$ ${\hat{y}}_{t}^{\left(i\right)}\left(p\right)=\gamma^{\left(i\right)}{\hat{x}}_{t}^{\left(i\right)}\left(p\right)\beta^{\left(i\right)}$  **End for**


### 3.4. Data Augumentation

**Figure 5:**
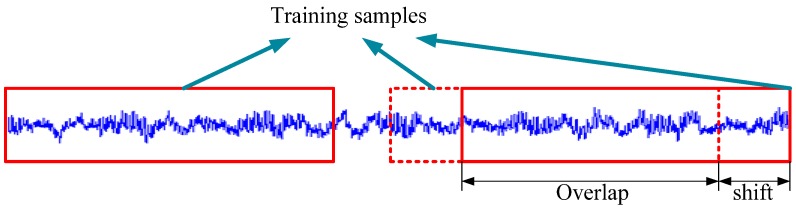
Data augmentation with overlap.

To acquire strong feature representations of the input raw signals, the WDCNN model is deep, and the first layer is wide, but this kind of structure could lead to the consequence that the model will easily get overfit without sufficient training samples. In computer vision, data augmentation is frequently used to increase the number of training samples to enhance the generalization performance of CNN [31]. Horizontal flips, random crops/scales, and color jitter are widely used to augment training samples in computer vision assignments. In fault diagnosis, data augmentation is also necessary for a convolutional neural network to achieve high classification precision. However, it is much easier to obtain huge amounts of data by slicing the training samples with overlap. This process is shown in Figure 5. The training samples is prepared with overlap. For example, a vibration signal with 60,000 points can provide the WDCNN with at most 57,953 training samples, each with a length of 2048 when the shift size is 1. Many papers overlook the effects of this simple operation, and most of these work use hundreds of training samples without any overlap [23,24]. In Section 4.3, we will validate the necessity of data augmentation.

## 4. Validation of the Proposed WDCNN Model

### 4.1. Data Description

**Figure 6:**
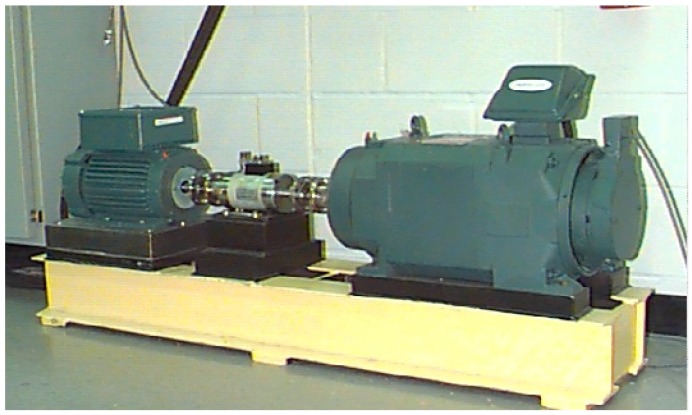
Motor driving mechanical system used by CWRU.

A large number of images need be prepared for image recognition tasks in order to use deep learning algorithms, which is especially true for CNNs. For example, the MNIST dataset contains 60,000 training data and 10,000 test data of handwritten digits. In order to train the CNN model sufficiently, we prepared a huge number of training samples. The original experiments data was obtained from the accelerometers of the motor driving mechanical system (Figure 6) at a sampling frequency of 12 kHz from the Case Western Reserve University (CWRU) Bearing Data Center [32]. There are four fault types of the bearing: normal, ball fault, inner race fault and out race fault. Each fault type contains fault diameters of 0.007 inch, 0.014 inch and 0.021 inch respectively, so we have ten fault conditions in total. In this experiment, each sample contains 2048 data points, which is easy to implement FFT for the baseline algorithm. Datasets A, B and C each contain 6600 training samples and 250 testing samples of ten different fault conditions under loads of 1, 2 and 3 hp. Dataset D contains 19,800 training data and 750 testing data of all three loads. In addition, the training samples are overlapped to augment data and there is no overlap among the test samples. The details of all the datasets are described in Table 1.

### 4.2. Experimental Setup

#### 4.2.1. Baseline System

We compare our methods with the deep neural network (DNN) [33] system with frequency features proposed by Lei et al. in 2016. The DNN system has two steps, namely unsupervised stacked auto-encoder pre-training and supervised fine tuning. This neural network consists of three hidden layers. The number of neurons in each layer is 1025, 500, 200, 100 and 10. The input of the network is the normalized 1025 Fourier coefficients transformed from the raw temporal signals using Fast Fourier transformation (FFT). Softmax is used as the classifier for supervised learning.

#### 4.2.2. Parameters of the Proposed CNN

The architecture of the proposed WDCNN used in experiments consists of five convolutional and pooling layers, then a fully-connected hidden layers, and at the end, a softmax layer. The size of first convolutional kernel is 64 × 1, and the rest kernel size is 3 × 1. The pooling type is max pooling and the activation function is ReLU. After each convolutional layer and fully-connected layer, batch normalization is used to improve the performance of WDCNN. In order to configure the parameters in the proposed model, some useful restrictions can be derived. The size of receptive field that each neuron in fully connected layer has on input signal is very important, which should be larger than at least one period of input, and smaller than the size of whole signal to be diagnosed. The iterative formula to compute the size of the receptive field of each neuron in the fully connected layer is:

(13) $$R^{\left(l-1\right)}=S^{\left(l\right)}(P^{\left(l\right)}R^{\left(l\right)}-1)+W^{\left(l\right)}$$

where *R*(*l*) denotes the size of receptive field that the first neuron in fully connected layer has in the *l*-th convolutional layer, *S*(*l*) denotes the compensation of *l*-th convolutional layer, *W*(*l*) denotes the size of convolutional kernel in *l*-th convolutional layer, and *P*(*l*) denotes the downsampling size of the *l*-th pooling layer.

As a result of the special architecture of WDCNN, when *l* > 1, *S*^(*l*)^ = 1, *P*^(*l*)^ = 2, *W*^(*l*)^ = 3, therefore *R*^(*l*+1)^ = 2*R*^(*l*)^ + 2, where *R*^(*n*+1)^ = 1, and *n* is the number of convolutional layers. Solving the iterative equation gives us:

(14) $$R^{\left(1\right)}=2^{n-1}\times 3-2$$

Therefore, the size *R*^(0)^ of the receptive field that each neuron in fully connected layer has on the input signal is:

(15) $$R^{\left(0\right)}=S^{\left(1\right)}(P^{\left(1\right)}R^{\left(1\right)}-1)+W^{\left(1\right)}=2S^{\left(1\right)}(2^{n-1}\times 3-2)+W^{\left(1\right)}-S^{\left(1\right)}\approx S^{\left(1\right)}(2^{n}\times 3-4)$$

To ensure the correct classification in the fully connected layer, the relation between the respective field *R*^(0)^ and the input signal should satisfy *T* ≤ *R*^(0)^ ≤ *L*, where *T* is the size of signal sampled in one rotating period, *L* is the size of the whole signal, in this paper, *T* ≈ 400, *L* = 2048. Besides, *L* must be divisible by *S*^(1)^. According to the rules above, we can easy find the stride for the first convolutional kernel is 8 or 16 when the number of convolutional layers is 5. When configuring parameters, it’s also worth noticing that with the increase of the number of convolutional kernels, width of kernels, depth of layers, and the decrease of stride, the number of neurons will increase, which improves the capacity of the model, but also makes it easier to overfit. Therefore, we need to enlarge the overlapping size between training signals to increase the number of training samples and vice versa.

The parameters of the convolutional and pooling layers are detailed in Table 2. The experiments were implemented using the Tensorflow toolbox [34] of Google. In order to minimize the loss function, the Adam Stochastic optimization algorithm is applied to train our CNN model. Adam is straightforward, memory-saving and computationally effective, which is quite suitable for models with large inputs data or many parameters. Details of this optimization algorithm can be found in [35].

### 4.3. Effect of the Data Number for Training

As a member of CNN, there are thousands of parameters in WDCNN. In order to suppress overfitting and enhance the generalization ability of WDCNN model, huge numbers of training samples are needed. To investigate how much data is sufficient and how well WDCNN can perform provided with enough training data, different sizes of training data is fed to train the network. In the following experiment, the performance of WDCNN is investigated using 90, 120, 300, 900, 1500, 3000, 6000, 12,000 and 19,800 training data, respectively. In fault diagnosis, the datasets can be balanced [36] or unbalanced [37], and whether the training dataset is balanced will cause differences in the evaluation method. As shown in Table 1, our dataset is a completely balanced dataset, which means accuracy is still an appropriate evaluation method to evaluate our algorithm in the following experiments.

**Figure 7:**
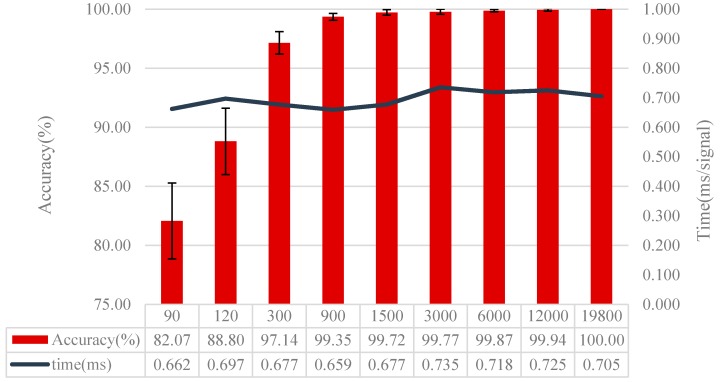
Diagnosis results using different numbers of training samples.

All the training samples are selected from the same signals, and the number of each fault type under different loads is the same. The samples from the first three training data sets have no overlap, and the rest have overlap. Twenty trials are conducted to alleviate the effects of random initial values of the network. We implemented the proposed method on an i7 6700 processor at 3.4 GHz with 16 GB memory. The test set is test set D in Table 2, and the results are shown in Figure 7.

**Figure 8:**
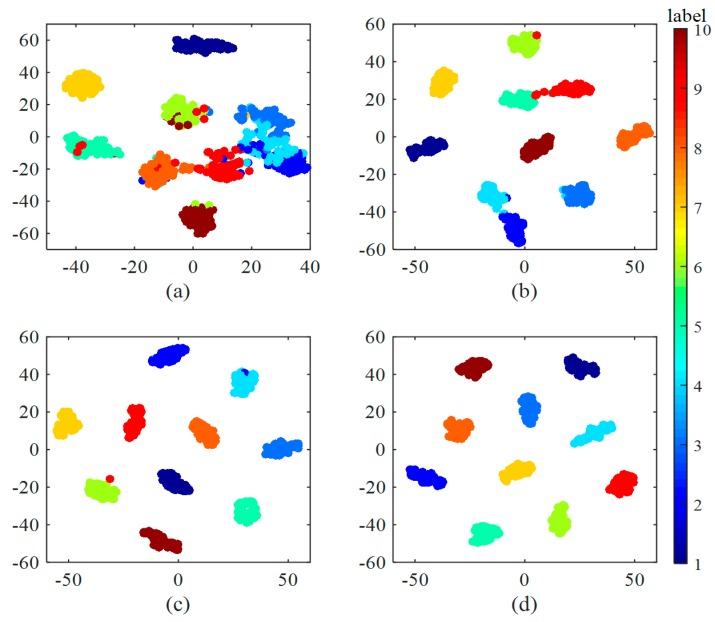
Feature visualization via t-SNE: last hidden fully-connected layer representation for the test samples in WDCNN trained by different numbers of training samples: (**a**) 90 training samples; (**b**) 300 training samples; (**c**) 3000 training samples and (**d**) 19,800 training samples.

In Figure 7, it is clear that the accuracy rises while its standard deviation declines with the increase of training samples. It shows that the accuracy increases by 15% when the training samples rise from 90 to 300. With 900 training samples, the accuracy is higher than 99%, reaching 99.35%. When the samples go up to 19,800, the accuracy peaks at 100% and the standard deviation is 0. It also can be seen from Figure 7 that after training with different size of training sets, it takes about 0.7 ms on average for the trained model to diagnose one signal sample, which satisfies our test time requirement. Besides, we can see that the increase of training samples almost has no influence on test time. To better understand the effect of training data, Figure 8 shows the last hidden fully-connected layer representation for the test samples in WDCNN trained by different numbers of training samples. From Figure 8, it is clear that the features are much more divisible with the increase of training samples, which enables the last layer softmax classifier easier to diagnose fault categories. This indicates that huge number of training data can make the proposed WDCNN more precise and stable because the increase of training samples can improve the generalization ability of the model [38], and it does not affect the time it needs to diagnose a signal sample. In the following experiments, the WDCNN model is trained with 19,800 samples.

### 4.4. Performance under Different Working Environment

In real world applications, the working environment of mechanical systems is very complicated. There are two main variations. First, the working load many change from time to time according to the production requisites, so it is unrealistic to collect and label enough training samples to make the classifier robust to all the working loads. Thus, it is significant for feature extractors and classifiers trained by samples collected in one working load to be able to learn and classify domain invariant features. Second, since the noise is unavoidable in industrial production, the vibration signals are easily contaminated by noise. The ability to diagnose the faults under noisy environment is crucial and challenging as well. In the reminder of this section, we will investigate how well the WDCNN method performs under these two scenarios.

#### 4.4.1. Case Study I: Performance across Different Load Domains

**Figure 9:**
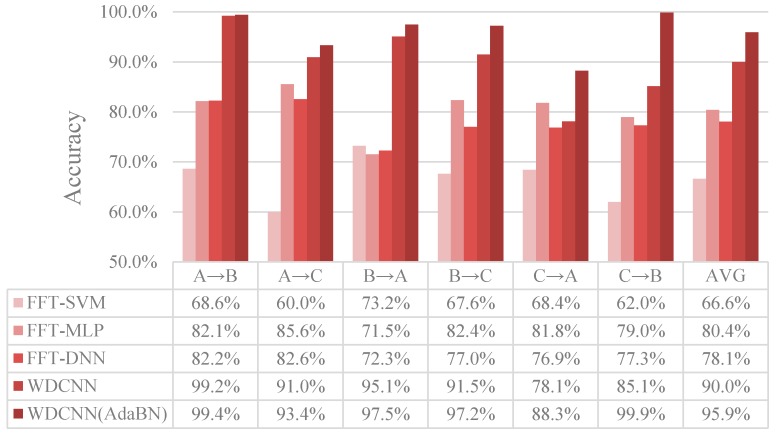
Results of the proposed WDCNN and WDCNN (AdaBN) of six domain shifts on the Datasets A, B and C, compared with FFT-SVM, FFT-MLP and FFT-DNN.

In this set of experiments, the adaptation performance across different load domains of WDCNN is tested and the domain adaptation algorithm AdaBN is used to improve the accuracy of the proposed WDCNN model. The results of our methods are compared with traditional SVM and MLP and the state of the art DNN algorithm which work in frequency domain (the data is transformed by FFT). The description of scenario settings for domain adaptation is illustrated in Table 3, and the results of the experiments are shown in Figure 9.

**Figure 10:**
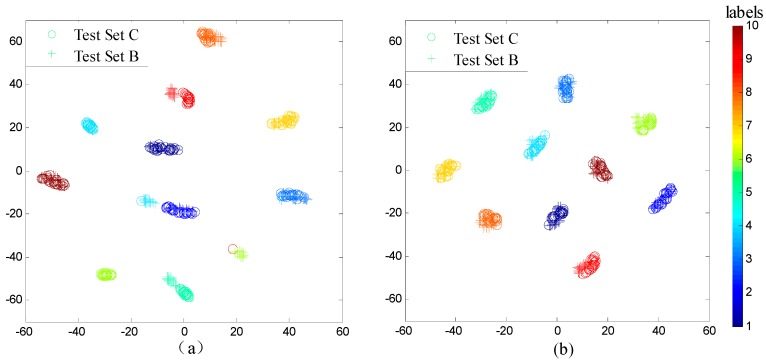
Feature visualization via t-SNE: last hidden fully-connected layer representation of WDCNN for (**a**) test set C and B before AdaBN, and (**b**) test C and B after AdaBN.

As shown in Figure 9, FFT-SVM, whose average accuracy in the six scenarios are under 70%, performs poorly in domain adaptation. MLP and DNN perform better, both achieving roughly 80% accuracy. In contrast, the proposed WDCNN method is much more precise than the compared algorithms, achieving 90.0% accuracy on average, which proves that the features learned by WDCNN from raw signals are more domain invariant than the traditional frequency features. Besides, with the help of AdaBN, the performance of WDCNN can be improved to 95.9%, close to the accuracy of WDCNN trained with 300 labeled data (Figure 7). In order to investigate the feature representation and why AdaBN can improve our WDCNN, we use t-SNE to visualize last hidden fully-connected layer representation for the test samples. With a well-trained WDCNN in dataset C (achieving 100% accuracy in test set C), we visualize the feature representation distribution of test sets C and B before AdaBN (shown in Figure 10a), and the feature representation distribution of tests C and B after AdaBN (shown in Figure 10b). It is clear that the feature representation distributions of test set B and test set C are not accordant before AdaBN, but right after the simple AdaBN, these two feature representation distributions are consistent with each other. In addition, there is no overlap between the fault feature representation between test sets B and C before AdaBN, which means the problem that leads to the low accuracy of WDCNN is caused by the last softmax layer, and that the feature representation itself is domain invariant.

#### 4.4.2. Case Study II: Performance under Noise Environment

In this case, we will discuss the diagnosis accuracy of the proposed WDCNN method and its domain adaptation algorithm. However, our scenario settings are different from the former validation case in which the SNR value of the training samples is 0 dB. In our experiments, the model is trained with the original data provided by CWRU, then it is tested with noisy data. This scenario better conforms with the conditions in realistic industrial production, because the noise varies a lot, and we can’t get all the labeled training samples under different noisy environment. First, we add additive white Gaussian noise to the original signals to composite signals with different SNR. The definition of SNR is shown as follows:

(16) $${\mathrm{SNR}}_{\mathrm{dB}}=10\log_{10}\left(\frac{P_{\mathrm{signal}}}{P_{\mathrm{noise}}}\right)$$

where *P*_signal_ and *P*_noise_ are the power of the signal and the noise, respectively.

**Figure 11:**
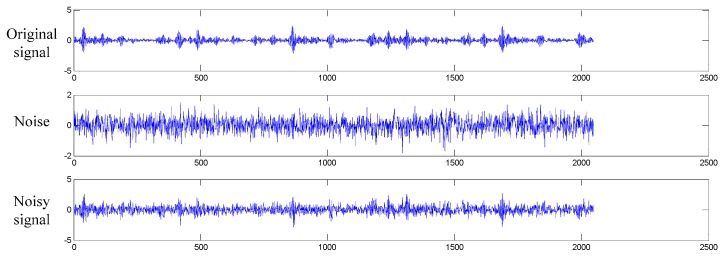
Figures for original signal of inner race fault, the additive white Gaussian noise, and the composite noisy signal with SNR = 0 dB respectively.

In Figure 11, the original signal of inner race fault is added with the additive white Gaussian noise. The SNR for the composite noisy signal is 0 dB, which means the power of noise is equal to that of the original signal. We test the proposed WDCNN model with noisy signals ranging from −4 dB to 10 dB. In order to verify the necessity of the first wide convolutional kernel, experiments are conducted with this width varying from 16 to 128, and the remaining structure of the network remaining unchanged.

**Figure 12:**
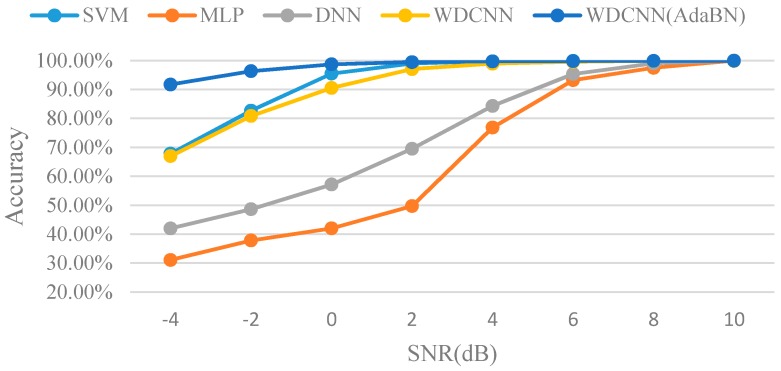
Comparison of classification accuracy under different noisy environment.

The results of the proposed WDCNN model without and with AdaBN diagnosing noisy signal are shown in Table 4 and Table 5. It is clear that the accuracy increases as the first-layer kernel becomes wider, e.g., the average accuracy is only 55.37% when the kernel size is 16, while the accuracy surges to 90.51% when the kernel size increases to 112. Besides, the best results occur at the kernel size of 104 and 112 instead of the max size 128, which also testifies that large kernel size is not suitable for extracting local features. In addition, WDCNN performs well when the noise is not very strong, and it can easily achieve over 99% accuracy when the SNR is over 4dB. In Table 5, it is interesting to find that WDCNN can reach over 90% accuracy even when SNR is −4 dB after AdaBN domain adaptation, and the minimum kernel size for achieving this accuracy is 40. The results were compared with SVM, MLP and DNN in Figure 12.

We can see that, WDCNN with AdaBN outperforms the other algorithms, and its denoising ability is almost the same as SVM, and much better than DNN. To sum it up, WDCNN model performs well under noisy environment without any denoising pre-processing.

### 4.5. Networks Visualizations

Generally, CNN is regarded as a black box, because it is hard to understand the inner operation mechanism of CNN, and few of the studies in fault diagnosis using CNN published so far have covered this topic. In this paper, we try to explore the inner operating process of the proposed WDCNN model by visualize the activations in this neural network.

**Figure 13:**
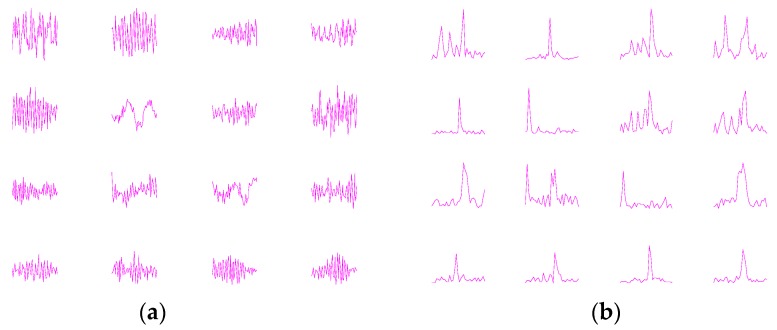
Visualization of convolutional kernels learned by (**a**) WDCNN and their (**b**) frequency-domain representation.

First, to better understand what kinds of features have been extracted by the fist-layer convolutional kernels, we plot the filter kernels learned by WDCNN and their frequency-domain representation. As shown in Figure 13a, the shapes of the filters vary a lot, and it is interesting to find that two filters (No. 6 and 11, from left to right and from upside to downside) are similar to the sinusoid waves. Figure 13b shows that all the filters focus on extracting intermediate and low frequency features, which helps reduce the impact of local high frequency features. Most of them concentrate on acquiring one or two frequency features, compared to FFT which extracts features of all frequency bands.

**Figure 14:**
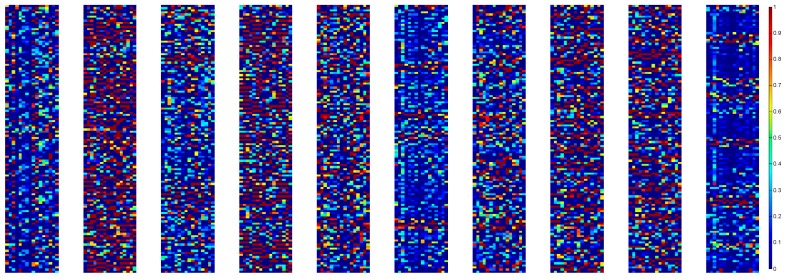
Visualization of the activations from the first convolutional layer with 10 kinds of fault signals as input. Red represents an activation of maximum, while blue means the neuron is not activated.

Next, we investigate the reactions of neurons in first convolutional layer when fed with different kinds of fault signals. Figure 14 shows the activations of input segment from 10 fault categories detailed in Table 1. Sixteen convolutional kernels transform the input 2048 × 1 signal into 128 × 16 maps, which are also called feature maps of CNN. In the feature map, the output neurons activated (the red ones) indicates a filter (convolutional kernel) matched strongly with the signal at the kernel receptive field. It can be seen that the feature maps are different for different fault type, which shows that the first-layer WDCNN can learn discriminant features from the raw signals. It is worth noticing that more neurons in the first convolutional layers are activated by fault signals compared with normal signals.

**Figure 15:**
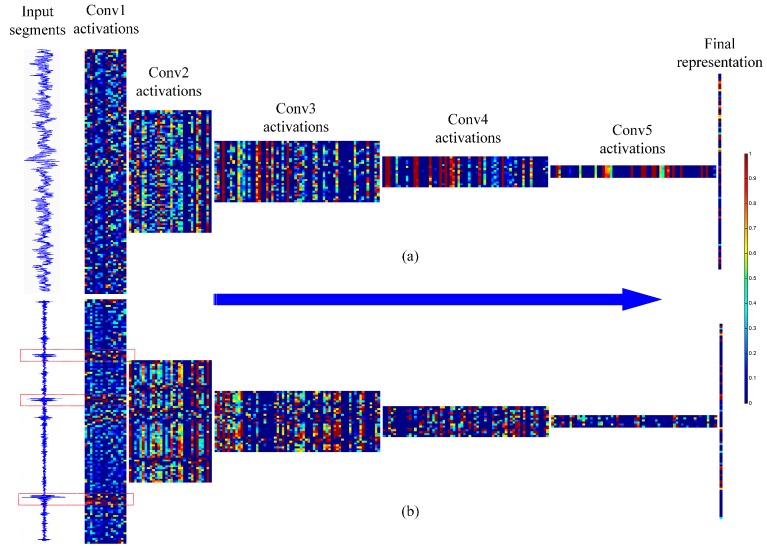
Visualization of all convolutional neuron activations in WDCNN for (**a**) a segment of normal vibration signal and (**b**) a segment of fault signal (inner race fault with 0.014-inch fault diameter). Red represents an activation of maximum, while blue means the neuron is not activated.

Thirdly, we visualize the reactions of neurons of all the convolutional layers to display what each layer “sees” in WDCNN. Two types of signals are chosen to make comparison, the normal fault and the inner race fault with 0.014-inch fault diameter. As shown in Figure 15, except for the first two layers, the size of feature map decreases as the layer goes deeper. The difference among feature maps of the same layer also increases as the convolutional layer goes deeper. People may have difficulty in discriminating the features in shallow layers, however it is easy to find out the difference between feature maps in the deep layers. The powerful discriminant features learned by deep layers indicates the reasonability to design a deep convolutional neural network.

**Figure 16:**
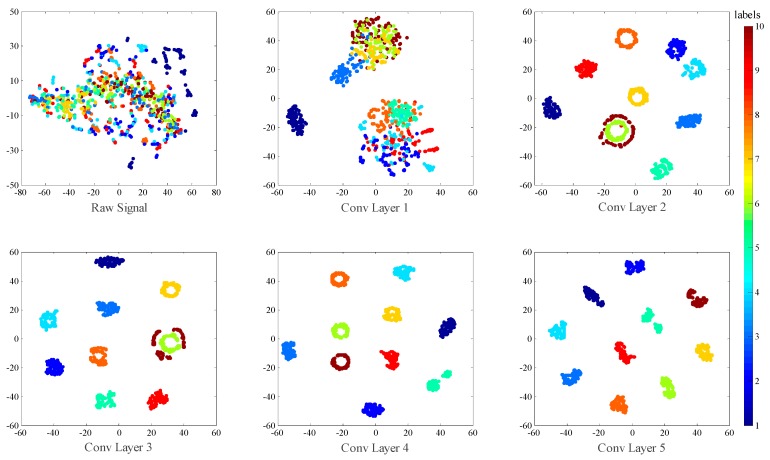
Visualization of the feature distribution of all the test samples with no noise extracted from each convolutional layers and the last fully-connected layer via t-SNE method.

Finally, t-SNE is used to investigate the feature distribution learned by each layer in Figure 16. There are some interesting phenomena worth noticing. First, we can notice that the normal class (C1) becomes dividable in Conv Layer 1, which suggests normal class is so easy to divide in our model that only one convolutional layer is enough to separate it out from the other classes. Second, as we can see in the visualization of Conv Layer 2, the feature points of C6 and C10 appears to be two concentric circles, with C10 surrounding C6. However, after two layers of Convolution operation, these two class become linearly dividable, as we can see in the visualization of Conv Layer 4. This shows the strong nonlinear mapping ability of convolutional neural networks.

## 5. Conclusions

This paper proposes a new model named WDCNN, to address the fault diagnosis problem. WDCNN works directly on raw vibration signals without any time-consuming hand-crafted feature extraction process. WDCNN has two main features, wide first-layer convolutional kernel and deep network structure with small convolutional layers. With the help of data augmentation, the proposed WDCNN model can easily achieve 100% accuracy in public CWRU bearing data set.

Results in Section 4 shows that, although state-of-the-art DNN model could achieve pretty high accuracy on normal datasets, its performance suffer from rapid degradation under noisy environment conditions or when the working load changes. However, WDCNN, with high classification accuracy, is also very robust to working load changes and noise.

When trained with data from one load domain, WDCNN can diagnose data from another load domain with high accuracy and the performance can be improved by an easy domain adaptation method named AdaBN. Besides, this model performs well under noisy environment conditions without any denoising pre-processing. In addition, network visualizations are used to investigate the inner mechanism of the proposed WDCNN model.

As mentioned in Section 4.3, the dataset used in this paper is completely balanced, while in practice, it’s possible to encounter unbalanced datasets. Therefore, in future work, we would investigate the performance of WDCNN on unbalanced dataset to expand the range of application of the algorithm.

It’s worth noticing that even though there is still room for improvement of WDCNN’s performance with the help of AdaBN, we should be aware that AdaBN requires statistical knowledge of the whole test data. However, in practice, only part of this knowledge is available, so we need to estimate the statistical information of the whole test data from the information of part of the data. This will make our algorithm more practical to use in real world applications.

Compared with traditional features, the features extracted by WDCNN are not easily influenced by environmental changes. Therefore, in future work, we can try using the first few layers as a feature extractor, and then train a classifier targeting to specific working environment, which may further improve the performance of the algorithm under different working environment.

## Figures and Tables

**Table 1:** Description of rolling element bearing datasets.

Fault Location		None	Ball			Inner Race			Outer Race			Load
Category Labels		1	2	3	4	5	6	7	8	9	10	
Fault diameter (inch)		0	0.007	0.014	0.021	0.007	0.014	0.021	0.007	0.014	0.021	
Dataset A no.	Train	660	660	660	660	660	660	660	660	660	660	1
	Test	25	25	25	25	25	25	25	25	25	25	
Dataset B no.	Train	660	660	660	660	660	660	660	660	660	660	2
	test	25	25	25	25	25	25	25	25	25	25	
Dataset C no.	train	660	660	660	660	660	660	660	660	660	660	3
	test	25	25	25	25	25	25	25	25	25	25	
Dataset D no.	Train	1980	1980	1980	1980	1980	1980	1980	1980	1980	1980	1,2,3
	Test	75	75	75	75	75	75	75	75	75	75	

**Table 2:** Details of proposed WDCNN model used in experiments.

No.	Layer Type	Kernel Size/Stride	Kernel Number	Output Size (Width × Depth)	Padding
1	Convolution1	64 × 1/16 × 1	16	128 × 16	Yes
2	Pooling1	2 × 1/2 × 1	16	64 × 16	No
3	Convolution2	3 × 1/1 × 1	32	64 × 32	Yes
4	Pooling2	2 × 1/2 × 1	32	32 × 32	No
5	Convolution3	3 × 1/1 × 1	64	32 × 64	Yes
6	Pooling3	2 × 1/2 × 1	64	16 × 64	No
7	Convolution4	3 × 1/1 × 1	64	16 × 64	Yes
8	Pooling4	2 × 1/2 × 1	64	8 × 64	No
9	Convolution5	3 × 1/1 × 1	64	6 × 64	No
10	Pooling5	2 × 1/2 × 1	64	3 × 64	No
11	Fully-connected	100	1	100 × 1	
12	Softmax	10	1	10	

**Table 3:** Description of scenario settings for domain adaptation.

Scenario Settings for Domain Adaptation			
Domain types	Source domain	Target domain	
Description	labeled signals under one single load	unlabeled signals under another load	
Domain details	Training set A	Test set B	Test set C
	Training set B	Test set C	Test set A
	Training set C	Test set A	Test set B
Target	Diagnose unlabeled vibration signals in target domain		

**Table 4:** Results for WDCNN under different noisy environment.

Kernel Size	SNR (dB)							
	−4	−2	0	2	4	6	8	10
**16**	27.14%	40.89%	55.37%	72.03%	85.71%	94.58%	98.41%	99.35%
**24**	35.32%	52.72%	70.15%	84.75%	94.37%	98.50%	99.64%	99.82%
**32**	42.00%	57.66%	72.76%	86.53%	95.47%	98.40%	99.52%	99.69%
**40**	46.84%	63.03%	77.55%	90.20%	97.07%	99.21%	99.71%	99.82%
**48**	50.15%	66.16%	80.21%	92.08%	97.69%	99.35%	99.73%	99.87%
**56**	51.67%	66.83%	80.85%	92.32%	97.84%	99.21%	99.73%	99.79%
**64**	51.75%	67.15%	82.03%	93.06%	98.07%	99.29%	99.79%	99.81%
**72**	53.69%	68.53%	82.23%	92.93%	97.91%	99.35%	99.71%	99.82%
**80**	56.07%	69.39%	84.24%	94.84%	98.69%	99.44%	99.83%	99.85%
**88**	56.05%	71.62%	85.33%	95.04%	98.46%	99.37%	99.74%	99.83%
**96**	64.29%	78.80%	89.91%	96.97%	99.03%	99.62%	99.81%	99.85%
**104**	62.91%	79.21%	90.36%	97.52%	99.23%	99.77%	99.81%	99.84%
**112**	66.95%	80.81%	90.51%	97.01%	98.88%	99.54%	99.83%	99.81%
**120**	61.84%	77.60%	90.47%	97.40%	99.08%	99.67%	99.81%	99.87%
**128**	60.88%	77.49%	89.79%	97.28%	99.13%	99.59%	99.83%	99.83%
**Max**	66.95%	80.81%	90.51%	97.52%	99.23%	99.77%	99.83%	99.87%

**Table 5:** Results for WDCNN with AdaBN under different noisy environment.

Kernel Size	SNR (dB)							
	−4	−2	0	2	4	6	8	10
**16**	81.84%	90.38%	95.66%	98.45%	99.01%	99.54%	99.75%	99.77%
**24**	87.24%	93.99%	97.34%	99.03%	99.61%	99.81%	99.87%	99.89%
**32**	89.81%	95.16%	97.93%	99.29%	99.55%	99.76%	99.77%	99.86%
**40**	90.96%	95.99%	98.32%	99.33%	99.59%	99.75%	99.83%	99.89%
**48**	91.69%	96.29%	98.33%	99.39%	99.67%	99.80%	99.81%	99.88%
**56**	92.65%	96.59%	98.61%	99.47%	**99.70%**	99.77%	99.86%	99.87%
**64**	92.56%	96.79%	**98.77%**	99.49%	99.67%	**99.83%**	99.87%	**99.93%**
**72**	92.36%	96.39%	98.51%	99.35%	99.61%	99.76%	99.79%	99.84%
**80**	92.31%	96.70%	98.67%	99.40%	99.62%	99.76%	99.87%	99.86%
**88**	92.61%	97.02%	**98.77%**	99.45%	99.63%	99.75%	99.81%	99.83%
**96**	**92.65%**	**97.04%**	**98.77%**	**99.57%**	99.67%	99.80%	99.83%	99.84%
**104**	92.45%	96.57%	98.63%	99.51%	99.67%	99.79%	99.81%	99.91%
**112**	91.70%	96.31%	98.68%	99.43%	99.67%	99.79%	**99.89%**	99.91%
**120**	92.11%	96.46%	98.75%	99.47%	99.66%	99.77%	99.83%	99.89%
**128**	91.92%	96.53%	98.67%	99.38%	99.63%	99.73%	99.82%	99.87%
**Max**	**92.65%**	**97.04%**	**98.77%**	**99.57%**	**99.70%**	**99.83%**	**99.89%**	**99.93%**

## Abbreviations

The following abbreviations are used in this manuscript:

CNN	Convolutional Neural Network
WDCNN	Deep Convolutional Neural Networks with Wide First-layer Kernels
AdaBN	Adaptive Batch Normalization
DNN	Deep Neural Network
SVM	Support Vector Machine
MLP	Multi-Layer Perceptron
ReLU	Rectified Linear Unit
BN	Batch Normalization
FFT	Fast Fourier Transformation
t-SNE	T-distributed Stochastic Neighbor Embedding
SNR	Signal-to-Noise Ratio
